# Long-latency auditory evoked potentials with verbal and nonverbal stimuli^☆^^☆☆^

**DOI:** 10.1016/j.bjorl.2014.10.005

**Published:** 2015-09-07

**Authors:** Sheila Jacques Oppitz, Dayane Domeneghini Didoné, Débora Durigon da Silva, Marjana Gois, Jordana Folgearini, Geise Corrêa Ferreira, Michele Vargas Garcia

**Affiliations:** a Human Communication Disorders, Universidade Federal de Santa Maria (UFSM), Santa Maria, RS, Brazil; b Universidade Federal de Santa Maria (UFSM), Santa Maria, RS, Brazil; c Fundo de Incentivo à Pesquisa (FIPE), Santa Maria, RS, Brazil; d Universidade Federal de São Paulo (UNIFESP), São Paulo, SP, Brazil

**Keywords:** Audiology, Electrophysiology, Evoked potentials, auditory, Event-related potentials, P300, Audiologia, Eletrofisiologia, Potenciais evocados auditivos, Potencial evocado P300

## Abstract

**Introduction:**

Long-latency auditory evoked potentials represent the cortical activity related to attention, memory, and auditory discrimination skills. Acoustic signal processing occurs differently between verbal and nonverbal stimuli, influencing the latency and amplitude patterns.

**Objective:**

To describe the latencies of the cortical potentials P1, N1, P2, N2, and P3, as well as P3 amplitude, with different speech stimuli and tone bursts, and to classify them in the presence and absence of these data.

**Methods:**

A total of 30 subjects with normal hearing were assessed, aged 18–32 years old, matched by gender. Nonverbal stimuli were used (tone burst; 1000 Hz – frequent and 4000 Hz – rare); and verbal (/ba/ – frequent; /ga/, /da/, and /di/ – rare).

**Results:**

Considering the component N2 for tone burst, the lowest latency found was 217.45 ms for the BA/DI stimulus; the highest latency found was 256.5 ms. For the P3 component, the shortest latency with tone burst stimuli was 298.7 with BA/GA stimuli, the highest, was 340 ms. For the P3 amplitude, there was no statistically significant difference among the different stimuli. For latencies of components P1, N1, P2, N2, P3, there were no statistical differences among them, regardless of the stimuli used.

**Conclusion:**

There was a difference in the latency of potentials N2 and P3 among the stimuli employed but no difference was observed for the P3 amplitude.

## Resumo

**Introdução:**

Os potenciais evocados auditivos de longa latência representam a atividade cortical relacionada às habilidades de atenção, memória e discriminação auditiva. O processamento do sinal acústico ocorre de maneira diferente entre estímulos verbais e não verbais, podendo interferir nos padrões de latência e amplitude.

**Objetivo:**

Descrever as latências dos potencias P1, N1, P2, N2 e P3 e a amplitude do P3 com os diferentes estímulos e classificar em presença e ausência estas informações.

**Método:**

Foram avaliados 30 indivíduos, com faixa etária de 18 a 32 anos. Equiparados quanto ao gênero e normo-ouvintes. Foram utilizados estímulos não verbais (1.000 HZ -frequente e 4.000 Hz -raro) e verbais (/ba/-frequente e /ga/, /da/, /di/-raros).

**Resultados:**

Considerando o componente N2, para o *tone burst* encontrou-se a menor latência em torno de 217,45 ms e para o estímulo BA/DI a maior latência em torno de 256,5 ms. No que diz respeito a componente P3, a latência encontrada com *tone burst* foi a menor em torno de 298,7 ms e com o estimulo BA/GA a maior em torno de 340 ms. Para a amplitude em P3, não houve diferença estatisticamente significante entre os diferentes estímulos. Quanto às informações referentes aos valores das latências dos componentes P1, N1, P2, N2 e P3, independente do estímulo utilizado houve presença dos componentes sem diferenças estatísticas entre eles.

**Conclusão:**

Houve diferença na latência do potencial N2 e P3 entre os estímulos, mas não foi observada diferença para a amplitude do P3.

## Introduction

Long-latency auditory evoked potentials (LLAEP) have been used in clinical practice to complement behavioral assessments of auditory processing. They are described as positive (P) and negative (N) peaks, which represent cortical activity related to attention, memory, and auditory discrimination skills.

The LLAEP include the positive 1 (P1), negative 1 (N1), positive 2 (P2), negative 2 (N2), and positive 3 (P3) waves, and are subdivided into exogenous potentials (P1, N1, P2, N2), which are influenced by the physical characteristics of the stimulus, such as intensity, duration, and frequency, and the endogenous potential (P3), predominantly influenced by the events related to cognitive skill.^1^

Frequent and rare stimuli (oddball paradigm) are used to obtain the cortical potentials. The most used stimuli in clinical practice are the tone burst, represented by a lower frequency (frequent stimulus) and a higher frequency (rare stimulus). However, a series of different stimuli, such as vowel, syllable, and word contrasts and even sentences can be used to evoke these potentials.^2^, ^3^

Some studies^4^, ^5^ have reported that acoustic signal processing occurs differently between verbal and non-verbal stimuli, which may influence the patterns of latency and amplitude of cortical potentials. Despite the lack of standardization of cortical potentials with speech stimuli, some studies indicate that these stimuli would be ideal for studying the neural basis of speech detection and discrimination,^3^, ^6^ and for contributing to additional information regarding complex signal processing.

Speech stimuli have been used to provide speech signal processing information in situations where behavioral assessment is not a precise method, helping in the identification of alterations in speech detection or discrimination.^7^

Based on the abovementioned facts and the need to characterize cortical potentials with different stimuli, the aim of this study was to compare the latency of cortical potentials P1, N1, P2, N2, and P3, as well as P3 amplitude, with different speech and tone burst stimuli.

## Methods

This study was approved by the Research Ethics Committee (REC) under protocol No. 25933514.1.0000.5346.

Individuals signed the informed consent, agreeing with the study objectives and participation.

A total of 30 individuals, aged 18–32 years, 15 females and 15 males, with normal hearing and no risk history for hearing, neurological, and language alterations were assessed.

The visual inspection of the external auditory canal was initially performed using a clinical Welch-Allyn otoscope to rule out any alterations that could influence audiometric thresholds.

Pure tone audiometry was performed in an acoustically treated booth, using a Madsen Itera II audiometer. Air conduction thresholds were assessed at the frequencies of 250, 500, 1000, 2000, 3000, 4000, 6000, and 8000 Hz, using the descending-ascending technique. Normal-hearing individuals were those with three-tone average (500, 1000, and 2000 Hz) ≤25 dB HL (decibel hearing level).^8^

Acoustic impedance measurements were performed using an Interacoustics AT235 middle ear analyzer to assess the tympanometric curve and acoustic reflexes. Reflexes were assessed at the frequencies 500–4000 Hz bilaterally in the contralateral mode. The sample included only individuals with type A tympanogram with present acoustic reflexes.^9^

Two-channel Intelligent Hearing Systems equipment was used for the detection of long-latency auditory evoked potentials. The skin was cleaned with abrasive paste and the electrodes were placed using electrolytic paste and adhesive tape, in the A1 (left mastoid), A2 (right mastoid), and Cz (vertex) positions, with the ground electrode (Fpz) placed on the forehead. The impedance value of the electrodes was required to be ≤3 kΩ.

The patient was instructed to pay attention to different stimuli (rare stimulus) that appeared randomly within a series of equal stimuli (frequent stimulus). The percentage of occurrence of rare stimuli was 20%, and 80% for frequent stimuli.

Non-verbal stimuli were used (tone burst) at the frequencies of 1000 Hz (frequent stimulus) and 4000 Hz (rare stimulus), as well as verbal stimuli (syllables /ba/ – frequent stimulus and /ga/, /da/, and /di/ – rare stimulus), presented binaurally at an intensity of 75 dB HL. For each type of stimulus (verbal/nonverbal) a total of 300 stimuli were used (approximately 240 frequent and 60 rare) to obtain the potentials. The tracings were not replicated, as replication can turn a rare stimulus into a frequent one for the patient. The parameters are described in Table 1.

**Table 1:** Mean and standard deviation for the P1, N1, P2, N2, and P3 components with all speech stimuli (BA-GA/BA-DA/BA-DI) and tone burst (1000 Hz × 4000 Hz).

Variables	Stimuli												*p*§
	BA × GA			BA × DA			BA × DI			1000 × 4000 Hz			
	*n*	Mean	SD	*n*	Mean	SD	*n*	Mean	SD	*n*	Mean	SD	
*P1*													
RE	26	62.2	8.1	27	59.8	8.1	25	65.5	18.3	22	62.2	11.9	0.393
LE	25	62.6	10.9	25	60.4	7.0	25	67.2	17.5	21	64.1	13.3	0.382
*p*-Value§	0.909			0.944			0.057			0.557			

*N1*													
RE	30	103.8^ab^	10.4	30	103.3^ab^	11.9	30	107.8ª	18.2	30	99.3^b^	14.7	0.038
LE	30	108.3	10.5	30	103.7	10.9	30	109.3	17.9	30	101.9	16.2	0.067
*p*-Value	<0.001			0.726			0.178			0.135			

*P2*													
RE	30	173.2^ab^	19.9	30	175.7^ab^	20.4	30	182.7ª	26.2	30	171.5^b^	26.7	0.026
LE	30	176.9^b^	17.0	30	175.5^b^	24.5	30	187.1ª	24.1	30	175.5^b^	28.6	0.017
*p*-Value	0.140			0.945			0.016			0.153			

*N2*													
RE	23	245.7^ab^	37.0	16	237.1^b^	43.4	14	251.6ª	37.7	10	216.4^c^	34.8	0.006
LE	22	255.3^ab^	29.6	14	232.6^b^	38.7	13	261.4ª	33.2	13	218.5c	39.2	0.003
*p*-Value	0.188			0.526			0.720			0.517			

*P3*													
RE	26	341.7^a^	44.2	26	301.5^c^	47.5	25	324.2b	59.2	25	297.0^b^	27.3	0.005
LE	26	344.4^a^	46.5	28	303.4^c^	46.3	21	329.9^ab^	63.4	24	300.4^b^	36.4	0.002
*p*-Value	0.171			0.325			0.619			0.163			

*Amplitude of P3*													
RE	27	6.2	2.2	30	6.9	5.3	24	6.3	2.8	26	5.8	2.1	0.208
LE	26	6.6^b^	2.1	28	7.8ª	5.4	21	6.7^b^	2.5	24	6.1^c^	2.3	0.027
*p*-Value	0.700			0.095			0.999			0.737			

§ Analysis of variance for repeated measures – *post hoc* Bonferroni, where means followed by the same letters (in line) do not differ significantly.

The study started with the pairs /ba/ and /ga/, followed by /ba/ and /di/, /ba/ and /da/, and tone burst, with all speech stimuli and tone burst presented prior to tracing, so that patients could become familiarized with the different stimuli. After the assessment of the first two speech stimuli, patients were instructed to rest, so that fatigue would not influence the answers of the last two sequences of stimuli.

Latency values were obtained by identifying the waves at the highest peak amplitude, with the P3 component considered only in the tracing of the rare stimuli, whereas P1, N1, P2, N2 were considered in the frequent stimulus, with no recorded reproduction of these waves, as the collection replication could result in fatigue and impair the assessment outcome, since it depends on the individual's attention.

Data were tabulated and statistically analyzed, comparing the latencies of components P1, N1, P2, N2, and P3 between speech stimuli and tone burst.

## Results

The results refer to the sample of 30 assessed individuals, with a mean age of 23.3 (± 3.5) years, with a minimum of 18 and maximum of 32 years. There was an equal gender distribution, with 50.0% (*n* = 15) for men and women.

Mean, and standard deviation measurements were obtained for the latency values of components P1, N1, P2, N2, and P3 as shown in Table 1.

For P1, N1 and P2 components, there were no significant differences detected between stimuli in both the RE and the LE.

For the N2 component, a significant difference was observed (*p*-value 0.006 and 0.003 for RE and LE respectively) for the latency measured in response to different stimuli, with the lower latency found for tone burst and the higher latency for the BA/DI stimulus.

Regarding the P3 component, there was a significant difference (*p*-value 0.005 and 0.002 for RE and LE, respectively) between the used stimuli and observed latency. The lowest latency was found with tone burst and the highest latency with the BA/GA stimulus.

There was no statistically significant difference among the different stimuli for the amplitude in P3.

As for the information regarding latency values of components P1, N1, P2, N2 and P3, for the four different stimuli, they were classified in the presence and absence of this information.

Regardless of the stimulus used, there were components with no statistical differences between them; Table 2 shows data for the absolute and relative distributions.

**Table 2:** Absolute distribution and relative to the presence and absence of information on the data for components P1, N1, P2, and N2 with all speech stimuli (BA–GA/BA–DA/BA–DI) and tone burst (1000 Hz × 4000 Hz).

Variables	Stimuli															
	BA × GA				BA × DA			BA × DI			1000 × 4000 Hz					
	Yes		No		Yes		No	Yes		No	Yes			No		
	*n*	%	*n*	%	*n*	%	*n*	%	*n*	%	*n*	%	*n*	%	*n*	%
*P1*																
RE	26	86.7	4	13.3	27	90.0	3	10.0	25	83.3	5	16.7	22	73.3	8	26.7
LE	25	83.3	5	16.7	25	83.3	5	16.7	25	83.3	5	16.7	21	70.0	9	30.0

*N1*																
RE	30	100.0	0	0.0	30	100.0	0	0.0	30	100.0	0	0.0	30	100.0	0	0.0
LE	30	100.0	0	0.0	30	100.0	0	0.0	30	100.0	0	0.0	30	100.0	0	0.0

*P2*																
RE	30	100.0	0	0.0	30	100.0	0	0.0	30	100.0	0	0.0	30	100.0	0	0.0
LE	30	100.0	0	0.0	30	100.0	0	0.0	30	100.0	0	0.0	30	100.0	0	0.0

*N2*																
RE	23	76.7	7	23.3	16	53.3	14	46.7	14	46.7	16	53.3	10	33.3	20	66.7
LE	22	73.3	8	26.7	14	46.7	16	53.3	13	43.3	17	56.7	13	43.3	17	56.7

*P3*																
RE	26	86.7	4	13.3	26	86.7	4	13.3	25	83.3	5	16.7	25	83.3	5	16.7
LE	26	86.7	4	13.3	28	93.3	2	6.7	21	70.0	9	30.0	24	80.0	6	20.0

*Amp P3*																
RE	27	90.0	3	10.0	30	100.0	0	0.0	24	80.0	6	20.0	26	86.7	4	13.3
LE	26	86.7	4	13.3	28	93.3	2	6.7	21	70.0	9	30.0	24	80.0	6	20.0

## Discussion

Despite the hemispheric differentiation and undeniable inequality in functional importance of the cerebral hemispheres, there were no differences between the performance of the right and left ears in the present study. Other studies have reported the absence of differences between ears,^10^, ^11^, ^12^ so the discussion will focus on the comparison between speech stimuli and tone burst, regarding the latency of exogenous components, and the latency and amplitude of the endogenous component P3.

In the present study, the component latencies, for P1, N1, and P2 revealed no differences in response to the four stimuli used (Table 1). Among the main endogenous components are the N2 and P3 waves, which showed differences in latency when the four stimuli were compared, being lower for both components with tone burst stimuli and higher for P3 with BA/GA stimulus, and even higher for N2 with BA/DI stimulus (Table 1).

This finding corroborates the study^10^ that reported that the stimulus used did not evoke any difference for the latency of components N1 and P2, but did influence the latency of components N2 and P3. This fact was expected, as the P3 component is a cognitive potential that is influenced by the stimulus and, therefore, these data are consistent with what has been previously reported in the literature.^11^, ^13^, ^14^

Regarding the comparison of speech and tone burst stimuli, the difference between them was expected, considering that the central activations are different for each stimulus, which corroborates the authors^10^ who reported that the type of stimulus used is an important variable in obtaining the N2 and P3 components. Verbal stimuli constitute a more difficult listening task when compared to non-verbal stimulus discrimination. Some authors^15^, ^16^ observed that the P3 latency increases when the “targets” for discrimination are more “difficult” than the standard, *i.e.*, latency is sensitive to the task processing demand.

This study showed that the speech stimulus influenced the N2 component, which has been observed by other authors,^17^ who mentioned that the N2 component registration appears to be related to the identification and attention processing of the rare stimulus, with a positive correlation between the value of its latency and the level of difficulty of the discrimination task. In one study^10^ the same fact was observed, where N2 was influenced by the speech stimuli and, in that study, the difference between stimuli was observed between vowel and consonant contrasts.

As for the amplitude, no difference was observed when comparing the stimuli.^7^, ^11^, ^13^, ^14^, ^18^ Some studies describe the reduction in amplitude of component P3 with the increased level of difficulty of the discrimination task. However, this correlation was not significant in the present study, which corroborates the findings of another study.^10^ The amplitude of potential P3 has been described as having great variability in the literature,^19^, ^20^, ^21^ and the normal range for the P300 amplitude is between 1.7 μV and 19.0 μV.

In this study, it was possible to obtain the records of the cortical and cognitive auditory evoked potentials P3 with speech stimulus with good producibility and morphology, demonstrating this is a viable procedure to be applied in clinical practice. This information was also reported by another author.^1^ All assessed components were observed with the four different stimuli in this study (Table 2), showing that for young adults, the morphological characteristics of the waves, as well as the presence of components do not depend on the type of stimulus to be elicited.

Nevertheless, it is known that the cognitive auditory evoked potential P3 generated by speech stimuli can also be used to provide information on speech signal processing, which according to the author^11^ helps to identify changes in detection or discrimination – information that can guide an individual's therapeutic rehabilitation.

The BA/GA stimulus brings more difficulty in syllable discrimination due to its proximity, when compared, for instance, to BA/DI syllables. Thus, this study makes an important contribution to the clinical and research areas, helping the professional choose the most appropriate stimulus for the subject to be assessed.

## Conclusion

There was a difference in latency of N2 and P3 potentials between the stimuli used; however, no difference was observed for the P3 amplitude.

## Conflicts of interest

The authors declare no conflicts of interest.
